# Analysis of Transcriptomic Differences in the Ovaries of High- and Low-Laying Ducks

**DOI:** 10.3390/genes15020181

**Published:** 2024-01-29

**Authors:** Yuguang Chang, Rongbing Guo, Tao Zeng, Hanxue Sun, Yong Tian, Xue Han, Yongqing Cao, Ligen Xu, Mingcai Duan, Lizhi Lu, Li Chen

**Affiliations:** 1 State Key Laboratory for Managing Biotic and Chemical Threats to the Quality and Safety of Agro-Products, Institute of Animal Science & Veterinary, Zhejiang Academy of Agricultural Sciences, Hangzhou 310021, China; changyuguang2023@126.com (Y.C.); zengtao4009@126.com (T.Z.); sunhx@zaas.ac.cn (H.S.); tyong@zaas.ac.cn (Y.T.); yongqingevo@163.com (Y.C.); xlg17855804562@163.com (L.X.); duanmc17@163.com (M.D.); lulizhibox@163.com (L.L.); 2 College of Animal Sciences and Veterinary Medicine, Zhejiang A&F University, Hangzhou 311300, China; guorongbing1234@163.com; 3 Institute of Animal Husbandry and Veterinary Science, Guizhou Academy of Agricultural Sciences, Guiyang 550000, China; hanxue8855601@126.com

**Keywords:** duck, ovary, RNA-seq, egg production

## Abstract

The egg-laying performance of Shan Ma ducks (Anas Platyrhynchos) is a crucial economic trait. Nevertheless, limited research has been conducted on the egg-laying performance of this species. We examined routine blood indicators and observed higher levels of metabolic and immune-related factors in the high-egg-production group compared with the low-egg-production group. Furthermore, we explored the ovarian transcriptome of both high- and low-egg-production groups of Shan Ma ducks using Illumina NovaSeq 6000 sequencing. A total of 1357 differentially expressed genes (DEGs) were identified, with 686 down-regulated and 671 up-regulated in the high-egg-production (HEP) ducks and low-egg-production (LEP) ducks. Several genes involved in the regulation of ovarian development, including neuropeptide Y (NPY), cell cycle protein-dependent kinase 1 (CDK1), and transcription factor 1 (E2F1), exhibited significant differential expressions at varying stages of egg production. Pathway functional analysis revealed that the DEGs were primarily associated with the steroid biosynthesis pathway, and the neuroactive ligand–receptor interaction pathway exhibited higher activity in the HEP group compared to the LEP group. This study offers valuable information about and novel insights into high egg production.

## 1. Introduction

Ducks, being the second-largest poultry species, significantly contribute to the economic income of poultry farmers, and the egg production rate plays an important role. The ovary, as a crucial organ for poultry egg-laying, plays a significant role in egg production [1]. Further research indicates that the process of folliculogenesis in the ovary determines the egg-laying performance of ducks [2]. RNA-seq technology has been applied to study the ovarian transcriptome of individuals with high- and low-egg-laying performances in poultry species, such as chickens, ducks, and geese [3,4,5]. Transcriptome sequencing technology has facilitated the identification of key candidate genes distinguishing between high- and low-egg-laying groups in poultry. For instance, Zhang and colleagues [6] screened five candidate genes (P2, WNT4, AMH, IGF1, and CYP17A1) associated with egg production in their analysis of the ovarian transcriptome of Jinghai yellow chickens exhibiting either comparatively high or low egg production.

The Shan Ma duck, recognized as an excellent egg-laying breed in Fujian Province, China, exhibits notable advantages, including strong adaptability [7]. Characterized by early maturity and a high-egg-laying rate, the Shan Ma duck typically starts laying eggs at an average age of 109 days, with a first-egg weight of 49.6 ± 3.7 g. By 300 days of age, the average egg production of Shan Ma ducks can reach 161 ± 15.0 eggs [8]. Although limited knowledge exists regarding the transcriptome analysis of genes associated with the egg production performance, the available information is scarce, with minimal subsequent validation, and there is little understanding of the linkages between genes. This research aimed to discover potential key genes related to high- and low-egg-laying rates in the ovarian tissue of the Shan Ma duck (*Anas platyrhynchos*).

## 2. Materials and Methods

### 2.1. Animals and Sample Collection

All the experimental procedures were reviewed and approved by the Institutional Animal Care and Use Committee at the Zhejiang Academy of Agricultural Science (Permit Number: 2022ZAASLA59). Shan Ma ducks were obtained from Zhoukou Guiliu Breeding Duck Breeding Co., Zhoukou, Henan, China. To maintain consistent conditions and ensure that the egg production performance was not affected, ducks of the same age and batch were carefully selected and fed under identical conditions. Each duck was randomly housed in individual cages and fed twice daily with free access to water. In this experiment, 315 ducks were fed and monitored from 120 days to 350 days (a total of 231 days), representing the highest egg-laying age stage for ducks. From the pool of 315 ducks, two groups were selected and categorized into high- and low-laying groups, each consisting of six ducks. The ducks with an egg production of higher than 207 eggs were considered as high egg production (HEP) and an egg production of lower than 180 eggs were considered as low egg production (LEP). The average egg production rate was 159 ± 21 for LEP ducks and 215 ± 8 for HEP ducks. Both groups underwent tests for egg quality, including the egg weight, eggshell strength, Haugh unit, and egg shape index [9]. The egg weight was measured using an electronic scale accurate to 0.01 g. The eggshell force gauge (Robotmation, Tokyo, Japan) was utilized to measure the eggshell breaking strength. The Haugh unit and egg yolk color were measured using an egg analyzer (Model: EA-01, ORKA, Israel). The eggshell thickness was measured using the eggshell thickness gauge (KARL DEUTSCH/ ETG-1061A). The egg shape index was measured using a vernier caliper accurate to 0.01 mm. The two groups of ducks were quickly killed by inhaling carbon dioxide and performing a cervical dislocation after fasting for about 12 h. Eight ovarian tissues (with the removal of hierarchical follicles and preservation of ovarian stroma) from 12 ducks (n = 4) were promptly placed into lyophilization tubes and stored at −80 °C for subsequent RNA extraction.

### 2.2. Serum Physiological and Biochemical Index Testing

In the HEP and LEP groups of the Shan Ma ducks, blood was collected from the brachial vein under the wing using a disposable blood collection needle (*n* = 4). Blood was collected in the early morning prior to the ducks’ feeding. During blood collection, the needle was inserted flat into the vein using the index and middle fingers. The collected blood was then placed in a vacuum blood collection tube without an anticoagulant (2 mL) and centrifuged at 3000 r/min for 10 min to obtain serum. The serum was dispensed into 1.5 mL centrifuge tubes and stored at −20 °C for analysis. The concentrations of follicle-stimulating hormone (FSH), luteinizing hormone (LH), estradiol (E2), growth hormone (GH), and prolactin (PRL), as well as the total protein (TP), albumin (ALB), globulin (GLOB), total cholesterol (TC), triglycerides (TG), alanine transaminase (ALT), aspartate transaminase (AST), high-density lipoprotein (HDL), and low-density lipoprotein (LDL) in the serum were determined using kits in an automatic biochemistry analyzer (all from the Beijing Sino-UK Institute of Biological Technology). Plasma concentrations were measured using ELISA and colorimetry.

### 2.3. RNA Extraction, Library Preparation, and Illumina Hiseq Sequencing

The total RNA was extracted from the ovarian tissues of 8 ducks using TRIzol^®^ reagent, following the manufacturer’s instructions (Invitrogen). Genomic DNA was then removed using DNase I (TaKara, San Jose, CA, USA). Then, the RNA quality was determined using a 2100 Bioanalyzer (Agilent, Santa Clara, CA, USA) and ND-2000 (NanoDrop Technologies, Wilmington, DE, USA). A high-quality RNA sample (OD260/280 = 1.8–2.2; OD260/230 ≥ 2.0; RIN ≥ 6.5; 28S:18S ≥ 1.0; >10 μg) was used to construct a sequencing library. 

RNA-seq transcriptome libraries were prepared using the TruSeqTM RNA sample preparation kit from Illumina (San Diego, CA, USA), including RNA isolation, RNA fragmentation, cDNA synthesis, end repair, A-base addition, and the ligation of the Illumina-indexed adaptors. The libraries were then size-selected for cDNA target fragments on low-range ultra agarose, followed by PCR amplification using Phusion DNA polymerase (NEB) for 15 PCR cycles. After quantification using TBS380, paired-end libraries were sequenced using Illumina NovaSeq 6000 sequencing (150 bp*2; Shanghai BIOZERON Co., Ltd., Shanghai, China). The RNA-seq data was submitted to the CNCB (China National Center for Bioinformation). The accession number is PRJCA016268.

### 2.4. RNA-Seq Data Processing and Analysis

The raw paired-end reads were trimmed and quality-controlled using Trimmomatic with parameters (SLIDINGWINDOW:4:15 MINLEN:75) (version 0.36). Subsequently, clean reads were aligned to the reference genome (https://www.ncbi.nlm.nih.gov/assembly/GCF_015476345.1 accessed on 14 November 2022) of Anas platyrhynchos using hisat2_v2.2.1 software. The quality of these data were assessed using qualimap_v2.2.1. HTSeq was used to count the reads for each gene, and the expression level of each gene was then calculated using the fragments per kilobase of exon per million mapped reads (FPKM) method. The R statistical package edgeR (Empirical analysis of Digital Gene Expression in R) was employed for differential expression analysis. The DEGs between two samples were selected using the following criteria: the logarithmic of fold change is greater than 2, and the false discovery rate (FDR) should be less than 0.05.

### 2.5. Gene Ontology and KEGG Pathway Analysis of DEGs

To comprehend the functions of the differentially expressed genes, GO functional enrichment, and KEGG pathway analysis were conducted using Goatools (https://github.com/tanghaibao/Goatools accessed on 14 November 2022) and KOBAS (http://kobas.cbi.pku.edu.cn/home.do accessed on 14 November 2022).

### 2.6. Protein–Protein Interaction Network Analysis and Module Selection

The Search Tool for the Retrieval of Interacting Genes (STRING) database was utilized to obtain protein–protein interaction (PPI) data. DEGs meeting the cutoff criteria, based on a significantly enriched pathway in the KEGG pathway (*p* < 0.05), were mapped to STRING to assess their interactive relationships. The PPI network of the cutoff DEGs was visualized using Cytoscape (ver. 3.9.1; http://www.cytoscape.org/ accessed on 14 November 2022). The Molecular Complex Detection (MCODE) app was employed to explore the modules within the PPI network.

### 2.7. Quantitative Real-Time PCR Analysis

To ensure the accuracy and reproducibility of the RNA-Seq data for two groups consisting of eight Shan Ma duck individuals from LEP and HEP, we randomly selected six differentially expressed genes (DEGs): NGFR, IGFBP3, GLUL, GSN, ZP1, and AMH. The RNA-Seq database was validated through a comparison with the qRT-PCR results for the DEGs, with a reference to β-actin. The primer fragment was designed using Primer Premier 5 software (Premier Biosoft, Palo Alto, CA, USA). The qRT-PCR test was performed in triplicate using TransScript one-step qRT-PCR Supermix (Transgen, Beijing, China) and run on a StepOnePlus real-time PCR system (Applied Biosystems, Carlsbad, CA, USA) under the following amplification conditions: 95 °C for 15 min, followed by 40 cycles at 95 °C for 15 s, 65 °C for 20 s, and 72 °C for 20 s. The relative quantification of the gene expression was performed using the 2^−ΔΔCt^ method.

### 2.8. Statistical Analysis 

All the independent-sample *T*-tests were conducted using SPSS, version 26.0 (IBM, Armonk, NY, USA). The results were expressed as the mean ± standard deviation. Differences were tested at a level of significance of *p* < 0.05 with the *t*-statistic. The correlation analysis was conducted using Pearson’s method.

## 3. Results

### 3.1. Egg Quality Differences between the HEP and LEP Groups

The egg production, eggshell thickness, egg weight, ovary weight, egg shape index, egg yolk color, eggshell strength, and Haugh unit were compared between the HEP and LEP groups (Table 1). Consistent with the screening model, the HEP group exhibited a 13% greater (*p* < 0.01) egg weight and a 9% greater (*p* < 0.01) Haugh unit than the LEP group. However, no significant differences were observed between the groups for the eggshell thickness, ovary weight, egg shape index, egg yolk color, and eggshell strength. 

### 3.2. Serum Physiological and Biochemical Differences between the HEP and LEP Groups

Table 2 illustrates the differences between the serum physiological and biochemical levels in the HEP and LEP groups. In the HEP group, the levels of FSH, LH, E2, GH, TP, GLOB, TC, TG, LDL, ALT, and ALB were significantly higher than those in the LEP group (*p* < 0.05). In the HEP group, the levels of P4 and HDL were significantly lower compared with those in the LEP group (*p* < 0.05). The AST level showed no statistically significant difference between the HEP and LEP groups (*p* > 0.05).

### 3.3. Transcriptome Alignment and Mapping Statistics

For this study, 8 cDNA libraries were constructed based on Shan Ma duck ovaries. The numbers of raw reads and clean reads of each library were more than 40 million each, except for L3, which had 39.6 million raw reads and 35.7 million clean reads. The GC content of all the samples ranged from 49.70% to 51.75%, with the base percentage of the Q20 exceeding 98.27% and the percentage of the Q30 base surpassing 94.51% (Supplementary Table S1). The ratio of the clean mapped reads to the reference genome for all the samples ranged from 82.66 to 88.42% (Supplementary Table S2). In summary, the sequencing data were suitable for the subsequent data analysis.

### 3.4. Differentially Expressed Genes (DEG) between the HEP and LEP Groups

**Figure 1:**
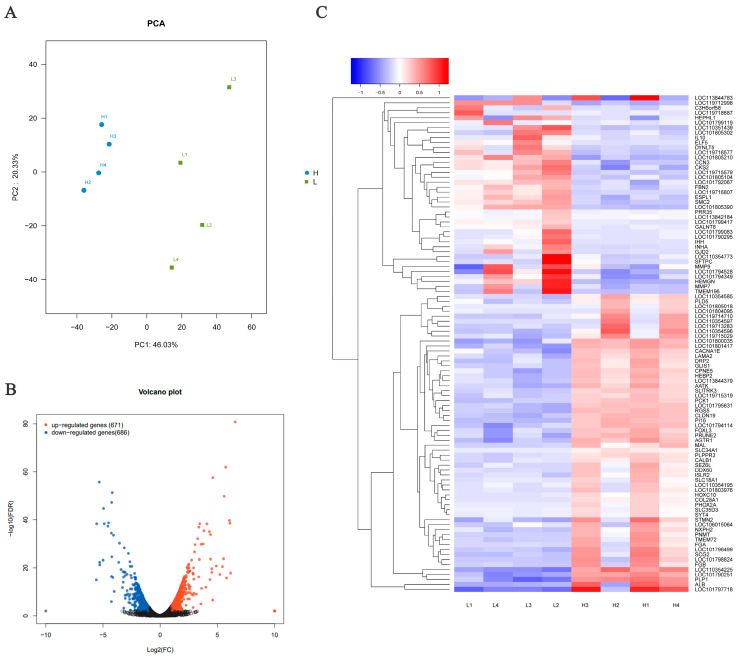
Ovarian gene expression differed between the HEP and LEP groups. (**A**) Principal component analysis graph. Each point in the diagram represents a sample, and the position of the sample in space is determined by the differences in expression of the genes contained within it. (**B**) Volcano plots of differential gene expression. Each point in the graph represents a specific gene or transcript, with red points indicating significantly up-regulated genes, blue points indicating significantly down-regulated genes, and black points indicating non-significantly different genes. (**C**) Heat map of DEGs. The color represents the level of expression: the redder the color, the higher the gene expression. The heat map in the analysis results is a plot of the top 100 genes with the smallest *p*-values for display.

Principal component analysis (PCA) was initially applied to analyze the samples. In general, the samples from different groups were separated into two distinct clusters in the PCA score plots, with a greater concentration within each group, indicating a noticeable difference between the LEP and HEP groups (Figure 1A). A total of 1357 genes were found to be differentially expressed between the two groups, comprising 671 up-regulated genes and 686 down-regulated genes (Figure 1B). The DEGs were further analyzed using hierarchical clustering analysis. Samples from the same group were clustered together, and the heatmap visually depicted the differences between the gene expression patterns in the LEP and HEP groups (Figure 1C). Twenty-six genes exhibited the highest up-regulation (log2 fold-change ≥ 4), while 50 genes showed the most significant down-regulation (log2 fold-change ≤ −4) among the 1357 differentially expressed genes (DEGs) identified in the HEP and LEP groups. The top 20 DEGs are listed in Table 3.

### 3.5. Gene Ontology (GO) and KEGG Analyses for DEGs

**Figure 2:**
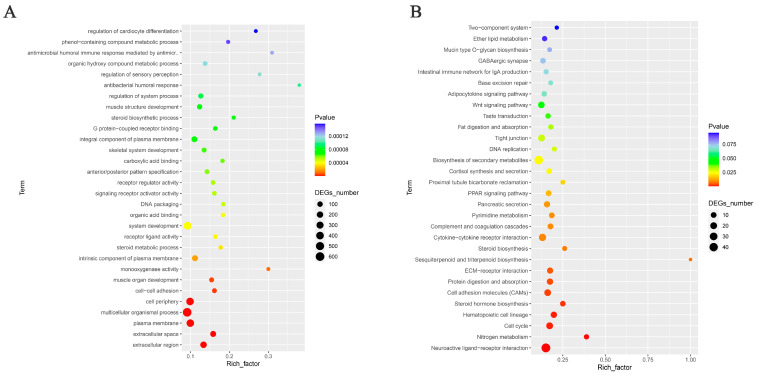
Functional annotations of differentially expressed genes in HEP and LEP groups. (**A**) Histogram of differential gene GO enrichment. The horizontal coordinate is the number of genes, and the vertical coordinate is the enrichment of genes in GO. (**B**) Scatter plot of KEGG enrichment for differentially expressed genes. The top 30 enrichment classifications of the KEGG pathway of the DEGs are listed in the figure. The horizontal axis indicates the enrichment factor, and the vertical axis indicates the name of the pathway. The point size indicates the number of enriched DEGs in the pathway, and the point color corresponds to a different range of *p*-values.

To delve deeper into the biochemical functions of the DEGs, we conducted KEGG pathway enrichment analysis and GO enrichment analysis. Out of the 1357 DEGs, 639 were enriched for the analysis (Figure 2A). Among them, 509 were enriched for biological processes (BPs), 56 for cellular components (CCs), and 74 for molecular functions (MFs). The main enrichment in biological processes included system development and multicellular organismal processes. Cellular components were primarily enriched in the cell periphery, plasma membrane, extracellular region, and integral components of the plasma membrane. Molecular functions showed significant enrichment in the G protein-coupled receptor binding, receptor regulator activity, and signaling receptor activator activity.

The KEGG enrichment analysis of the top 30 pathways is presented in Figure 2B. The figure illustrates that differentially expressed genes are significantly involved in the neuroactive ligand–receptor interactions, cytokine–cytokine receptor interactions, biosynthesis of secondary metabolites, cell adhesion molecules (CAMs), ECM–receptor interactions, protein digestion and absorption, and hematopoietic cell lineage.

### 3.6. Identification of Hub Genes and Pathways through PPI Network Analysis of DEGs

**Figure 3:**
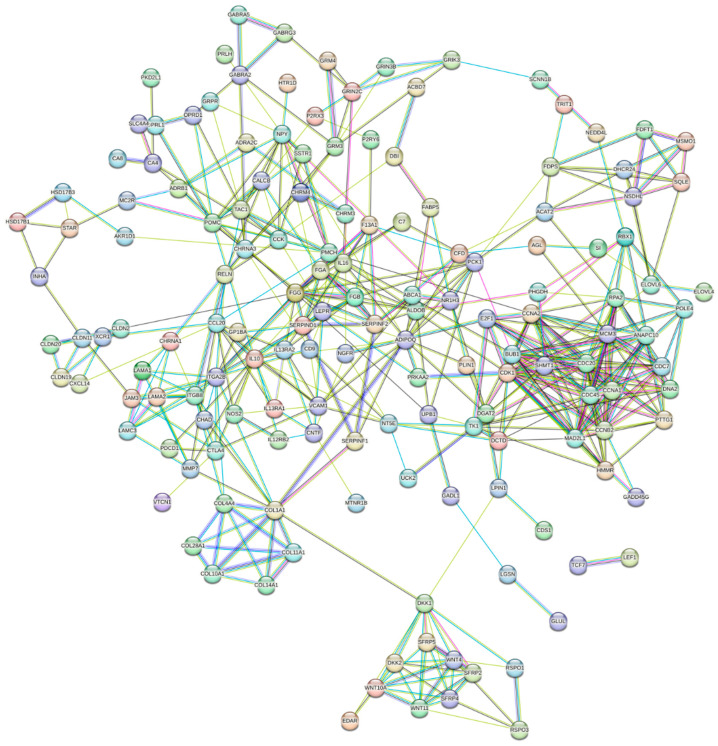
Protein–protein interaction (PPI) network for the cutoff differentially expressed genes (DEGs) based on the KEGG pathway. A total of 170 nodes and 450 edges were identified. The line color indicates the type of interaction evidence.

**Figure 4:**
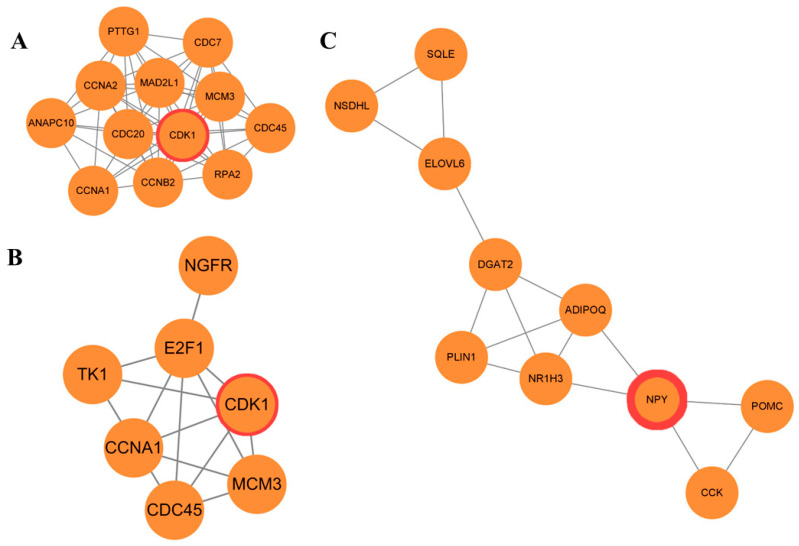
The three protein–protein interaction (PPI) hub network modules. The three significant modules, including (**A**) module 1 (MCODE score = 3.3), (**B**) module 2 (score = 10), and (**C**) module 3 (score = 3.7), were constructed from the PPI network of differentially expressed genes using MCODE. The seed node of each module was shaped, as highlighted by the red gene symbols.

The PPI network was constructed with 170 nodes and 450 edges (Figure 3). Using the STRING database, Cytoscape methods, and KEGG pathway analysis, we identified 3 key genes associated with the laying production performance. Additionally, 3 significant modules—module 1 (MCODE score = 3.3), module 2 (MCODE score = 10), and module 3 (MCODE score = 3.7)—were identified from the PPI network using the MCODE algorithm applied to the cutoff DEGs by MCODE (Figure 4). Module 1 (Figure 4A) consists of 10 nodes and 15 edges. Module 2 (Figure 4B) comprises 10 nodes and 15 edges. Module 3 (Figure 4C) includes 12 nodes and 55 edges.

### 3.7. Validation of RNA-Seq Results by qRT-PCR

**Figure 5:**
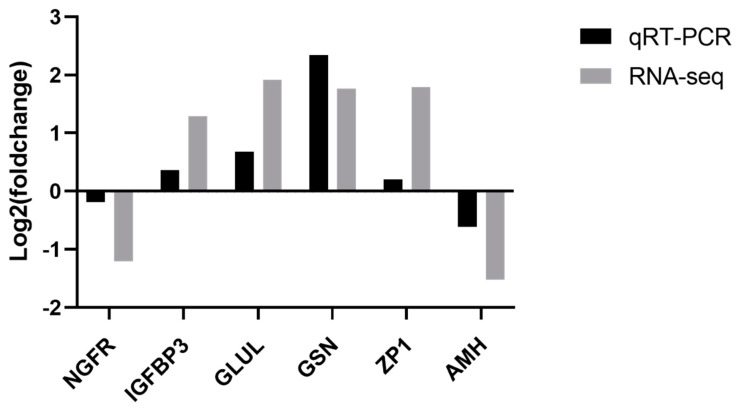
Comparative analysis of qRT-PCR versus RNA-seq. Selected DEGs were validated by qRT-PCR for comparison.

To validate the accuracy of the RNA-seq data, six DEGs were selected, comprising two down-regulated genes (NGFR and AMH) and four up-regulated genes (IGFBP3, GLUL, GSN, and ZP1) in the HEP versus LEP comparison. The results from the qPCR analysis of the six DEGs randomly selected from the transcriptomic sequence data revealed that the expression trend of these genes was consistent with the transcriptome sequencing data (see Figure 5), indicating the reliability of the RNA-seq results.

## 4. Discussion

### 4.1. Differences between Egg Quality in High- and Low-Egg-Production Shan Ma Ducks

The number of eggs laid and the egg quality are pivotal indicators of ducks’ egg production performance, offering valuable guidance for duck selection and improvement [10]. The egg quality and egg production are influenced by a multitude of factors, including age, light intensity, nutritional elements, and genetic factors [11,12,13]. This study, conducted through a comparison of high- and low-egg-producing groups, reveals a significant increase in both the egg weight and Haugh unit within the HEP group compared with the LEP group. However, no statistically significant differences were observed between the two groups regarding other egg quality indices, including the eggshell thickness, egg shape index, egg yolk color, and eggshell strength.

Elevated egg weight typically indicates improved egg quality [14]. Prior research has demonstrated that incorporating certain organic substances can enhance the feed utilization, consequently improving the egg quality [15]. This could explain why the high-yielding group in this experiment exhibited superior feed utilization, resulting in higher egg weights. 

The Haugh unit, serving as a metric for the egg white quality, represents the freshness of the egg. The egg white, rich in various bioactive proteins, is beneficial to human health [16]. The feed conversion ratio, defined as the ratio of the feed intake to the egg production, is intricately linked to the gut microbial composition, subsequently influencing the nutrient absorption [17,18]. These studies propose that high-egg-laying groups with elevated Haugh units may exhibit an enhanced nutrient absorption capacity along with increased microbial presence

### 4.2. Blood Physiological and Biochemical Indicators

The endocrine system plays an important role in regulating the egg production performance of poultry. The egg production performance in female poultry is primarily influenced by the growth and development of ovarian follicles, and egg production is related to the frequency of ovulation, which is mainly controlled by the hypothalamic–pituitary–gonadal (HPG) axis, and daily ovulation is necessary for producing progesterone and LH [19,20]. GH is a protein secreted by the pituitary gland of animals and can improve energy metabolism, increase the ovarian growth efficiency, and accelerate growth [21,22]. Increased GH levels elevate LDL and cholesterol in the blood, as well as insulin-like growth factor I (IGF-I) in the ovary, thereby stimulating follicular development, consistent with the results of this experiment [23,24]. FSH promotes follicle maturation and stimulates P4 secretion, while LH promotes follicular maturation and triggers ovulation [25]. In this experiment, FSH and LH were increased in the HEP group and could promote follicular maturation and, thus, increase egg production. Higher levels of estradiol in the blood of high-yielding turkeys are consistent with the present results, and it is possible that estradiol reduces the occurrence of follicular atresia, which in turn increases egg production [19,26]. PRL inhibits gonadotropins, leading to follicular atresia and even failure to ovulate, thus negatively regulating the reproductive activity [27]. Therefore, the HEP group exhibited a reduced occurrence of follicular atresia and increased egg production via lowered PRL levels. The experimental results demonstrated significantly higher levels of T3, T4, FSH, LH, E2, and GH in the HEP group. This elevation promoted the growth and development of follicles, consequently leading to increased egg production. 

As protein indicators, serum TP, ALB, and GLOB levels impact protein transport, metabolism, and immunity and exhibit a positive correlation with egg production [28,29]. The experimental results revealed that the serum TP, ALB, and GLOB levels were significantly higher in the HEP group than in the LEP group, suggesting superior protein utilization in the HEP group.

TC and TG are lipids in the blood; TC is involved in lipid absorption and metabolism, while TG is responsible for functions and energy storage, showing a positive correlation with egg production [29,30]. Cholesterol and LDL are crucial for egg production, and an elevation in HDL leads to a decrease in LDL, influencing egg production [31]. The experimental results suggest that the HEP group exhibited enhanced egg production via improved lipid metabolism. ALT and AST are involved in protein synthesis and metabolism in animals. ALT levels show a positive correlation with the number of eggs produced, while AST is not significantly correlated with egg production [29,32]. The experimental results aligned with expectations, indicating higher protein synthesis and metabolism in the HEP group.

### 4.3. Analysis of DEGs

In this experiment, the sequencing of the ovarian tissues of high- and low-egg-production groups of Shan Ma ducks revealed enrichment in 1357 differentially expressed genes. Identifying the differentially expressed genes (DEGs) related to reproduction involved KEGG analysis, followed by protein–protein interaction network analysis, leading to the discovery of three DEGs. The three genes (NPY, CDK1, and E2F1) were associated with signaling pathways related to reproductive performance, including cytokine–cytokine receptor interactions and neuroactive ligand–receptor interactions and the cAMP signaling pathway, TGF-β signaling pathway, nitrogen metabolism, p53 signaling pathway, and PI3K-Akt signaling pathway.

NPY has been implicated in neuroactive ligand–receptor interactions that act on both the hypothalamus and the anterior pituitary gland, influencing the LH release and modulating reproductive hormone secretion [33,34]. At the cellular level, NPY can induce granulosa cell proliferation, and its production varies with the stage of follicular development [35]. In conclusion, NPY may potentially affect the egg production performance in multiple ways by regulating hormone levels and granulosa cell proliferation.

CDK1 is a kinase that regulates the mitotic G2/M phase transition, and its deletion leads to the premature senescence of granulosa cells [36,37,38]. In the study of geese, CDK1 was possibly responsible for the regulation of follicular atresia, which is an important factor affecting egg production [39]. In this study, the low expression of CDK1 in the HEP group may improve the egg production performance by delaying the senescence of granulosa cells.

E2F1 is a transcription factor involved in the cell cycle and apoptosis, driven by growth factors and cytokines to different cell fates. It also regulates the expression of growth factors and cytokine receptors, establishing positive and negative feedback mechanisms [40]. E2F1 is primarily expressed in the G1/S phase of the cell cycle, inducing the transcription of various genes for DNA synthesis. It is considered as essential for cells’ entry to the S phase and the subsequent progression of cell cycle proteins [41]. Whether E2F1 promotes apoptosis or proliferation depends mainly on its binding to growth factors or apoptotic factors. In functional studies of porcine ovarian granulosa cells, reduced E2F1 promotes the expression of key genes for follicular development and estrogen synthesis, while reducing granulosa cell apoptosis [42]. The down-regulation of E2F1 in the ovarian tissues of the high-yielding Shan Ma ducks in this experiment may have inhibited granulosa cell apoptosis and promoted ovarian development.

### 4.4. Functional Classification Analysis

To comprehend the distinctions in the physiological functions of differentially expressed genes in the ovaries of high- and low-egg-production Shan Ma ducks, the identified genes underwent GO and KEGG enrichment analyses. The GO enrichment analysis revealed that the differentially expressed genes in the cellular fractions of the high- and low-egg-production Shan Ma ducks were predominantly concentrated in the peripheral, plasma membrane, and extracellular regions. These regions primarily facilitate the binding and regulation of various hormones to receptors, playing a crucial role in regulating the developmental capacity of ovarian follicles [43]. In terms of molecular functions, differentially expressed genes are predominantly enriched in G protein-coupled receptor binding and receptor regulator activities. These pathways exert regulatory effects on the endocrine system and are pivotal for ovulation and follicular development in the ovary [44]. As an example, G protein-coupled receptors engage in crosstalk with epidermal growth factor (EGF), forming a network that utilizes EGF family members as mediators of LH action in the ovulating follicle. This, in turn, regulates ovulatory ovulation [45].

The analysis of the KEGG-signaling pathways revealed that the differentially expressed genes in the high- and low-laying Shan Ma ducks were predominantly involved in the neuroactive ligand–receptor interactions, cytokine–cytokine receptor interactions, secondary metabolite biosynthesis, cell adhesion molecules (CAMs), ECM–receptor interactions, protein digestion and uptake, and hematopoietic cell lineage. These pathways might be associated with the reproductive performance of the Shan Ma duck. Notably, CAMs and ECM–receptor interactions were significantly enriched. These gene-enriched pathways have previously demonstrated involvement in the regulation of egg-laying genes in other poultry studies. [46,47]. Among them, CAMs and ECM–receptor interactions were significantly enriched. They directly or indirectly regulate stem cell proliferation through multiple mechanisms [48]. CAMs are present on the surface of all cells, binding to receptors on the extracellular ECM or other cells. They act not only as signaling factors but also as signaling receptors, transducing signals triggered by cellular interactions. This, in turn, regulates various processes, such as cell division, migration, and differentiation, contributing to maintaining a stable tissue structure [49]. Furthermore, CAMs in the circulatory system regulate physiological functions in vascular homeostasis and innate and adaptive immune responses [50]. However, during atherosclerosis, CAMs play a role in facilitating the process, primarily by modulating the inflammatory response and endothelial function, as well as the ability to drive plaque rupture. All these factors contribute to atherosclerotic progression [51]. ECMs are macromolecules secreted by cells into the extracellular mesenchyme. They have a highly dynamic structure, are present in all tissues, and are essential for life. ECMs interact with cells and regulate various functions, including cell proliferation, migration, and differentiation. Mutations in the gene encoding the ECM can result in embryonic lethality [52]. Moreover, the ECM interacts with epithelial cells as a ligand for cell receptors and transmits a variety of regulatory signals. These signals include those regulating cell adhesion, migration, proliferation, and apoptosis, as well as isolating and locally releasing growth factors [53]. Ovarian follicles are situated in an avascular environment where oocytes and granulosa cells are interconnected by gaps. They communicate mainly by adhesion to regulate ovulation and the growth and development of follicles in this ovary [54]. Hence, the hypothesis is that cell adhesion factors and extracellular matrix interactions regulate ovarian follicular development.

## 5. Conclusions

There are many biological factors that regulate egg production. At the level of the reproductive hormones, it was found that elevated levels of FSH, LH, E2, and GH are correlated with increased egg production; at the level of the biochemical indicators, higher levels of TP, ALB, GLOB, TC, TG, LDL, and ALT are associated with increased egg production. Transcriptome analysis revealed 1357 significant DEGs in the HEP and LEP groups. GO analysis indicated that the genes predominantly participate in the peripheral, plasma membrane, and extracellular regions, with functions including G protein-coupled receptor binding and receptor regulator activities. KEGG analysis indicated the predominant involvement of some genes in pathways, such as the neuroactive ligand–receptor interactions, cytokine–cytokine receptor interactions, secondary metabolite biosynthesis, cell adhesion molecules (CAMs), ECM–receptor interactions, protein digestion and uptake, and hematopoietic cell lineage. These findings suggest the pathways and crucial hormones regulating poultry egg production, offering valuable functional genes for further research on enhancing poultry yields.

## Figures and Tables

**Table 1:** Egg production and egg quality results of Shan Ma ducks in high-egg-production (HEP) and low-egg-production (LEP) groups.

Item	LEP	HEP	*p*-Value
Egg production	184.33 ± 4.55	215.50 ± 13.52 **	0.002
Eggshell thickness (mm)	0.44 ± 0.02	0.42 ± 0.04	0.069
Egg weight (g)	69.53 ± 7.43	78.55 ± 7.43 **	0.008
Ovary weight (g)	2.58 ± 0.95	3.03 ± 1.08	0.784
Egg shape index	1.31 ± 0.06	1.32 ± 0.06	0.279
Egg yolk color	11.25 ± 0.45	11.36 ± 0.50	0.909
Eggshell strength (kg/cm^2^)	4.74 ± 0.37	4.77 ± 0.79	0.151
Hastelloy unit	63.87 ± 17.86	69.93 ± 12.36	0.002

Note: “**” represents highly significant difference (*p* < 0.01), and no asterisk represents insignificant difference.

**Table 2:** Differences between serum physiological and biochemical index levels in LEP and HEP groups.

Item	LEP	HEP	*p*-Value
Follicle-stimulating hormone (mIU/mL)	1.77 ± 0.26	3.07 ± 0.19 **	0.000
Luteinizing hormone (mIU/mL)	5.64 ± 0.83	8.22 ± 1.44 *	0.021
Estradiol (pg/mL)	31.55 ± 6.14	47.20 ± 5.45 **	0.009
Progesterone (ng/mL)	12.12 ± 0.92	8.26 ± 0.90 **	0.001
Growth hormone (ng/mL)	4.70 ± 0.72	7.06 ± 0.56 *	0.002
Prolactin (μIU/mL)	99.80 ± 2.86	87.12 ± 6.46 **	0.012
Triiodothyronine (ng/mL)	0.73 ± 0.17	1.06 ± 0.09 *	0.015
Thyroxine (ng/mL)	9.14 ± 0.64	12.71 ± 1.09 **	0.001
Total protein (g/L)	37.51 ± 5.99	57.55 ± 7.35 **	0.006
Albumin (g/L)	13.15 ± 1.01	15.81 ± 0.88 **	0.007
Globulin (g/L)	32.03 ± 1.25	36.24 ± 2.49 *	0.023
Total cholesterol (mmol/L)	1.98 ± 0.10	3.65 ± 0.38 **	0.000
Triglycerides (mmol/L)	9.30 ± 1.95	17.49 ± 2.75 **	0.003
High-density lipoprotein (mmol/L)	1.74 ± 0.50	0.90 ± 0.14 *	0.019
Low-density lipoprotein (mmol/L)	0.88 ± 0.19	1.16 ± 0.11 *	0.045
Aspartate transaminase (U/L)	48.90 ± 7.19	115.96 ± 56.41	0.056
Alanine transaminase (U/L)	60.99 ± 5.25	82.22 ± 6.71 **	0.002

Note: “*” represents significant difference (*p* < 0.05), “**” represents highly significant difference (*p* < 0.01), and no asterisk represents insignificant difference.

**Table 3:** The top 20 DEGs.

Gene ID	Read Count (LEP)	Read Count (HEP)	Log2 Fold-Change (HEP/LEP)	Up/Down (HEP/LEP)	*p*-Value	Gene Description
LOC101797718	0.58	54.78	6.55	up	0.00	Apovitellenin-1-like precursor
LOC101805018	0.00	0.86	9.76	up	0.00	Prostatic acid phosphatase-like
MAL	0.03	2.03	6.16	up	0.00	Myelin and lymphocyte protein
LOC110354597	0.00	2.13	11.05	up	0.00	Cygnin
FGA	0.03	2.12	6.10	up	0.00	Fibrinogen alpha chain
FGB	0.05	3.07	6.04	up	0.00	Fibrinogen beta chain
ALB	0.20	10.51	5.73	up	0.00	Serum albumin precursor
LOC101793493	0.00	1.46	10.51	up	0.00	Fatty acid-binding protein, liver
PHOX2A	0.01	0.79	5.80	up	0.00	Paired mesoderm homeobox Protein 2A
PNMT	0.00	2.20	11.10	up	0.00	Phenylethanolamine N-methyltransferase
LOC110351439	5.40	0.14	−5.28	down	0.00	Histone H3
LOC101799083	1.67	0.03	−5.58	down	0.00	Trefoil factor 2
LOC119716807	2.07	0.06	−5.00	down	0.00	Putative short transient receptor potential channel 2-like protein
TMEM196	9.33	0.23	−5.32	down	0.00	Transmembrane protein 196 isoform X3
GJD2	2.05	0.07	−4.95	down	0.00	Gap junction delta-2 protein
IHH	1.96	0.04	−5.55	down	0.00	Indian hedgehog protein
LOC119718687	1.86	0.05	−5.29	down	0.00	Butyrophilin subfamily 1 member A1-like
SFTPC	6.33	0.21	−4.89	down	0.00	Pulmonary surfactant-associated protein C isoform X1
LOC110354773	7.78	0.33	−4.57	down	0.00	Pulmonary surfactant-associated protein C-like
LOC119715269	0.40	0.00	−8.66	down	0.00	Trypsin-3-like

Note: The top 20 DEGs are listed in the table, including the functional descriptions of the genes.

## Data Availability

All the original resequencing data used in this study have been submitted to the public database of the CNCB (China National Center for Bioinformation). The accession number is PRJCA016268. All the other presented data are available in either the main manuscript or the Supplementary Materials.

## Supplementary Materials

The following supporting information can be downloaded at https://www.mdpi.com/article/10.3390/genes15020181/s1: Table S1: Sequencing data quality testing; Table S2: Comparison of reads with the reference genome.
