# Supplementary material for: Analysis of Transcriptomic Differences in the Ovaries of High- and Low-Laying Ducks
Source: Genes (Basel). 2024 Jan 29;15(2):181. doi: 10.3390/genes15020181 (PMC10887599; doi:10.3390/genes15020181)
Supplement: Supplementary file 1 [file genes-15-00181-s001.zip › Supplementary Table S1.pdf]

Supplementary Table S1. Sequencing data quality testing.

Sample	Raw_Reads	Clean_Reads	Q20%	Q30%	GC%
L1	40915694	40687106	98.49	95.11	49.9
L2	42744516	42465692	98.28	94.51	50.81
L3	39681906	35749998	98.81	96.12	49.7
L4	49369112	49016808	98.49	95.16	51.75
H1	41345888	41067350	98.82	96.19	49.98
H2	51531560	51052494	98.63	95.63	49.94
H3	42650294	42373518	98.62	95.55	50.24
H4	47073112	46694818	98.63	95.59	50.08

Abbreviations: L, low egg production; H, high egg production.
