# Supplementary material for: Analysis of Transcriptomic Differences in the Ovaries of High- and Low-Laying Ducks
Source: Genes (Basel). 2024 Jan 29;15(2):181. doi: 10.3390/genes15020181 (PMC10887599; doi:10.3390/genes15020181)
Supplement: Supplementary file 1 [file genes-15-00181-s001.zip › Supplementary Table S2.pdf]

Supplementary Table S2. Comparison of Reads with the reference genome.

Sample	clean-reads	mapped-reads	mapped-rate(%)
L1	40687106	35873821	88.17
L2	42465692	37548164	88.42
L3	35749998	29690373	83.05
L4	49016808	42571097	86.85
H1	41067350	35350774	86.08
H2	51052494	42199991	82.66
H3	42373518	35801385	84.49
H4	46694818	38766037	83.02

Abbreviations: L, low egg production; H, high egg production.
